# A Comparison of Self-Reported and Objective Physical Activity Measures in Young Australian Women

**DOI:** 10.2196/publichealth.4259

**Published:** 2015-10-05

**Authors:** Stefanie Hartley, Suzanne Garland, Elisa Young, Kim Louise Bennell, Ilona Tay, Alexandra Gorelik, John Dennis Wark

**Affiliations:** ^1^ Murdoch Childrens Research Institute Parkville Australia; ^2^ The Royal Women's Hospital Department of Molecular Microbiology & Infectious Diseases Parkville Australia; ^3^ University of Melbourne Parkville Australia; ^4^ Melbourne Health Royal Melbourne Hospital Parkville Australia

**Keywords:** physical activity, exercise, women’s health, questionnaires

## Abstract

**Background:**

The evidence for beneficial effects of recommended levels of physical activity is overwhelming. However, 70% of Australians fail to meet these levels. In particular, physical activity participation by women falls sharply between ages 16 to 25 years. Further information about physical activity measures in young women is needed. Self-administered questionnaires are often used to measure physical activity given their ease of application, but known limitations, including recall bias, compromise the accuracy of data. Alternatives such as objective measures are commonly used to overcome this problem, but are more costly and time consuming.

**Objective:**

To compare the output between the Modified Active Australia Survey (MAAS), the International Physical Activity Questionnaire (IPAQ), and an objective physical activity measure—the SenseWear Armband (SWA)—to evaluate the test-retest reliability of the MAAS and to determine the acceptability of the SWA among young women.

**Methods:**

Young women from Victoria, Australia, aged 18 to 25 years who had participated in previous studies via Facebook advertising were recruited. Participants completed the two physical activity questionnaires online, immediately before and after wearing the armband for 7 consecutive days. Data from the SWA was blocked into 10-minute activity times. Follow-up IPAQ, MAAS, and SWA data were analyzed by comparing the total continuous and categorical activity scores, while concurrent validity of IPAQ and MAAS were analyzed by comparing follow-up scores. Test-retest reliability of MAAS was analyzed by comparing MAAS total physical activity scores at baseline and follow-up. Participants provided feedback in the follow-up questionnaire about their experience of wearing the armband to determine acceptability of the SWA. Data analyses included graphical (ie, Bland-Altman plot, scatterplot) and analytical (ie, canonical correlation, kappa statistic) methods to determine agreement between MAAS, IPAQ, and SWA data.

**Results:**

A total of 58 participants returned complete data. Comparisons between the MAAS and IPAQ questionnaires (n=52) showed moderate agreement for both categorical (kappa=.48, *P*<.001) and continuous data (*r*=.69, *P*<.001). Overall, the IPAQ tended to give higher scores. No significant correlation was observed between SWA and IPAQ or MAAS continuous data, for both minute-by-minute and blocked SWA data. The SWA tended to record lower scores than the questionnaires, suggesting participants tended to overreport their amount of physical activity. The test-retest analysis of MAAS showed moderate agreement for continuous outcomes (*r*=.44, *P*=.001). However, poor agreement was seen for categorical outcomes. The acceptability of the SWA to participants was high.

**Conclusions:**

Moderate agreement between the MAAS and IPAQ and moderate reliability of the MAAS indicates that the MAAS may be a suitable alternative to the IPAQ to assess total physical activity in young women, due to its shorter length and consequently lower participant burden. The SWA, and likely other monitoring devices, have the advantage over questionnaires of avoiding overreporting of self-reported physical activity, while being highly acceptable to participants.

## Introduction

There is overwhelming evidence of the health, social, and economic benefits of engaging in recommended levels of physical activity [1,2]. Physical activity guidelines for Australians are to accumulate 150-300 minutes of moderate-intensity physical activity, 75-150 minutes of vigorous-intensity physical activity, or an equivalent combination of both moderate and vigorous activities each week [3]. However, close to three-quarters of the Australian population aged 18-65 years fail to meet these levels [4], a finding common to other developed countries [5]. Research into interventions to increase physical activity levels in the community is therefore warranted. In particular, young women should be targeted for interventions, as women play a large role in influencing household activity levels and the amount of physical activity women engage in falls sharply during the ages of 16-25 years [6].

Physical activity is defined as "any bodily movement produced by skeletal muscles that requires energy expenditure" [7]. It can occur in various forms and contexts, such as actions performed during recreation, sports, work, household chores, and gardening. The health benefits of physical activity include prevention and management of chronic diseases as well as overall reduced mortality and improved mental health [1,2].

Given the importance of physical activity, valid and precise assessment of activity levels are needed to determine activity trends, explore associations between health and physical activity, predict population health outcomes, and evaluate the effectiveness of physical activity interventions [8,9]. However, it is a methodological challenge to accurately measure physical activity in individuals, due to the complexities of daily life [10] and the technical requirements to make meaningful measurements. The burden and inadequate precision of quantifying physical activity levels has created a market for the development of better measuring methods. Various methods to capture levels of physical activity in free-living individuals are available and are categorized under (1) subjective (self-reported) and (2) objective measures. For research purposes, it is important to select reliable and valid measures that can be feasibly administered to individuals.

Self-reported questionnaires, while low cost and simple to administer, can vary in accuracy due to recall bias, social desirability bias, and misinterpretation [11]. A systematic review of physical activity questionnaires recommended 23 questionnaires, identified as having good content validity [8]. One of these was the International Physical Activity Questionnaire (IPAQ), which is considered to be the most extensively validated questionnaire across 12 countries [12]. The Modified Active Australia Survey (MAAS) is a less commonly used questionnaire to measure physical activity [13], although it has been employed in large Australian national and state surveys [13,14]. The MAAS was developed by shortening the Active Australia Survey, and has been shown to have comparable reliability and validity to the Active Australia Survey [13]. Both the IPAQ and MAAS ask the respondent about the duration, frequency, and intensity of activity in the preceding 7 days, and include activities such as walking, sports, yard and housework, and bicycling. MAAS has the advantage of being considerably shorter than the long-form IPAQ and is relevant to the Australian context. When tested in middle-aged Australian women, it has also been found to have comparable reliability and validity to that reported for the full version of the Active Australia Survey [13]. However, neither the test-retest reliability of the MAAS nor its concurrent validity compared to other questionnaires, such as the widely accepted IPAQ, has been established in younger women.

A variety of noninvasive objective measures is commercially available to assess physical activity levels. One of these is the SenseWear Armband (SWA) activity monitor (Model: MF-SW) (BodyMedia, Inc, Pittsburgh, PA, USA), which is a small, lightweight, multisensor activity monitor worn on the upper arm. It integrates body motion and step count from a three-axis accelerometer while other sensors such as heat flux, galvanic skin responses, and skin surface temperature can provide other data. The SWA has been extensively validated in numerous peer-reviewed publications [15]. However, the SWA is costly and not waterproof, meaning that it cannot record water-based physical activity. To date, a comparison of measures from the SWA and the IPAQ and MAAS has not been conducted in young Australian women. It is also not known whether the SWA is an acceptable method of collecting physical activity measures in this population.

The primary aims of the study in young Australian women were to (1) compare the output from the SWA with self-reported measures of physical activity by IPAQ and MAAS and (2) determine the acceptability of using the SWA to assess levels of physical activity. The secondary aims were to evaluate (1) the concurrent validity of MAAS by comparing IPAQ and MAAS and (2) the test-retest reliability of MAAS.

## Methods

### Participants

This project was a substudy of the Young Female Health Initiative (YFHI), a comprehensive study of lifestyle, health, and well-being in young women aged 16-25 years living in the state of Victoria, Australia. This study received ethical approval from the Human Research and Ethics Committees of the Royal Women’s Hospital, Victoria, Australia.

Cross-recruitment of participants from previous studies, namely the YFHI pilot [16] and the Vaccine Against Cervical Cancer Impact aNd Effectiveness (VACCINE) studies [17], was the main recruitment method employed. The majority of women in these 2 studies had previously volunteered by responding to advertisements on Facebook. Women were approached for this study if they met the inclusion criteria and had consented to be contacted for future studies. Individuals who expressed an interest in the study from the YFHI website [18] and fulfilled the inclusion criteria also were recruited. The recruitment of 70 participants for this substudy took place from June to September 2012. All participants gave verbal and written informed consent for the study, after the nature and possible consequences were explained.

To be eligible for the substudy, participants needed to be YFHI study participants and to satisfy the following criteria: (1) female, (2) aged 16-25 years, (3) living in Victoria, Australia, (4) provide verbal and written consent, and (5) willing to complete 2 questionnaires and to wear an SWA for 7 consecutive days. Participants were excluded if they were living outside Victoria, were unable to give consent due to a language barrier, had a physical impairment, or if there were any other reasons that would affect the completion of the study.

### Procedure

Participants were emailed a link to the online baseline questionnaire containing the MAAS (16 items) and IPAQ (self-administered long-form version; 49 items), and a set of questions on acceptability of the SWA. Questionnaires were administered using the online survey tool, SurveyMonkey [19].

Participants were sent a study package by post containing an information and consent form, an SWA with instructions on its use, and an armband usage log. Participants were instructed to wear the armband on the back of the upper left arm (over the triceps muscle) for 7 consecutive days, removing it only for water-based activities, such as showering or swimming. They were also asked to record on the monitor log provided when the SWA was removed, entering details including the time the armband was removed, the time it was replaced, activities undertaken during that time, and the intensity level of any physical activity (low, moderate, or vigorous). Participants were asked to return the package via registered post, or deliver it directly to the study office.

Participants were emailed a link to the follow-up physical activity questionnaires as completion of the 7-day period wearing the SWA approached. These were identical to the baseline questionnaires, with the addition of feedback questions about the participant’s experience of wearing the SWA. Participants who completed the study were given AUD $10 in the form of a gift voucher to a retail store as minor compensation for their time.

### Analysis of Questionnaires

MAAS asks the respondent the duration, frequency, and intensity of activity in leisure time, household or garden chores, and sedentary behavior in the last 7 days. The MAAS questionnaires were analyzed and scored according to the method described in Brown et al [13]. Participants who scored "none" in the total physical activity were grouped in the *low* category, to enable comparisons with IPAQ. The IPAQ scoring protocol was used for the IPAQ [20]. In brief, for both questionnaires, participants are classified into low, medium, and high levels of activity based on specified rules for occasions and minutes of various-intensity exercise. As well as this categorical outcome, a continuous outcome can be calculated based on metabolic equivalent of task (MET) minutes per week. Both MAAS and IPAQ give scores for different domains and intensities of physical activity. These are then combined to give a total score. For this study, only the total scores of the MAAS and IPAQ were used in the analysis.

### Analysis of SenseWear Output

Returned devices were connected to a computer and minute-by-minute data were downloaded from the device using SenseWear Professional Software 7.0 (BodyMedia Inc, Pittsburgh, PA, USA). The data were used to estimate the frequency and duration of each activity as well as METs that are based on the participant’s gender, age, height, and body mass. The MET data were analyzed twice as follows: (1) using minute-by-minute data, and (2) using *blocked* data. Blocked data were generated to allow comparisons between the SWA data and the 2 questionnaires. The IPAQ and MAAS ask only about physical activity performed for 10 minutes or more, whereas the SWA collects minute-by-minute data. To enable more meaningful comparisons between the SWA and the questionnaires, SWA data were blocked into activity of 10 minutes or more. A similar method of blocking was used by Brown et al [13].

The following conditions were applied to generate blocked data: (1) the time between the first and last reading must be 10 minutes or longer, (2) the first and last times must show an MET ≥ 3.3, (3) for blocks of 15 minutes or less, 80% of MET values must be ≥ 3.3, (4) for blocks of over 15 minutes, 75% of MET values must be ≥ 3.3, (5) for blocks of 15 minutes or less, “rests” must be no longer than 2 minutes, (6) for blocks of over 15 minutes, “rests” must be no longer than 3 minutes, (7) for blocks of 30-60 minutes, there can be one 4-minute “rest," (8) for blocks over 60 minutes, there can be two 4-minute “rests,” and (9) rest was defined as an MET value of less than 3.3.

For our analyses, participants who had at least 4 monitoring days, including 2 weekend days, were included. A valid day was defined as having at least 1296 on-body minutes, after inclusion of known activities from the monitor log. This corresponds to 90% of a 24-hour period. Participants who fell short of the criteria were excluded from analysis.

### Calculation of Scores

**Figure 1:**
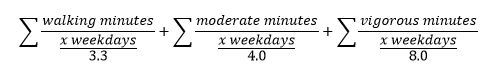
Equation used to calculate average weekday scores from the SenseWear Armband activity monitor.

Average weekday scores were obtained by using the equation shown in Figure 1, where *x* denotes the number of valid monitoring weekdays the participant has provided. A similar method was used to generate average weekend scores.

With the averages for both weekday and weekend scores, a weekly total score was then calculated using the following formula:

5 (average weekday score) + 2 (average weekend score)

### Statistical Analysis

Data analyses were performed using SPSS version 17.0 (SPSS Inc, Chicago, IL, USA) and STATA version 13.0 (StataCorp LP, College Station, TX, USA). Descriptive statistics were reported as n and percentages for categorical data, and mean (SD) for continuous data, except the interval between completion of baseline and follow-up questionnaires that was reported as median (interquartile range [IQR]). All continuous data were tested for normality prior to data analysis. Both MAAS and IPAQ scores had skewed distributions and were transformed using natural logarithmic transformation. MAAS and IPAQ were analyzed twice—as continuous and as categorical.

Pearson correlation was used to measure the association between total continuous outcomes of the self-reported questionnaires (IPAQ and MAAS) with the SWA. Calculation of MAAS test-retest reliability was only conducted for participants who indicated that their overall physical activity levels had not changed between completion of the baseline and follow-up questionnaire, due to wearing the SWA.

Standard analytical (ie, canonical correlation) and graphical (ie, scatterplot, Bland-Altman plot) techniques were used to determine the agreement between continuous measures—MAAS, IPAQ, and SWA scores—while kappa statistics were used to determine the agreement between categorical data—IPAQ and MAAS categories. In all cases, statistical significance was defined at *P*<.05.

## Results

### Participants

A total of 58 participants returned an SWA with data. Of these, 54 (93%) completed the baseline questionnaire and 52 (90%) completed the follow-up questionnaire. A total of 4 out of 58 (7%) were excluded as they had less than 4 days of recorded data. The mean days of SWA wear was 6.43 (SD 0.67; range 4-7) and the mean total time of SWA wear was 133.65 hours (SD 21.01; range 68.15-161.57). The mean age of participants was 22.1 years (SD 2.0; range 18.5-25.3). Demographic characteristics of participants are shown in Table 1.

**Table 1:** Demographic characteristics of participants (n=54).

Characteristic	Categories	n (%)^a^
**Age (years)**		
	18-21	26 (48)
	22-25	28 (52)
**Country of birth**		
	Australia	52 (96)
	Other	2 (4)
**Geographic region of residence**		
	Major city	45 (83)
	Inner regional	9 (17)
	Outer regional/remote	0 (0)
**Body mass index (kg/m** ^**2**^ **) (using self-reported height and weight)**		
	Underweight	6 (11)
	Normal	32 (59)
	Overweight	13 (24)
	Obese	3 (6)
	Extremely obese	0 (0)
**Education level** ^b^		
	<Year 12	4 (7)
	Year 12	19 (35)
	>Year 12	31 (57)
**Socioeconomic level (SEIFA** ^c^ **percentile)** ^d^		
	≤25 (most disadvantaged)	2 (4)
	26-100	52 (96)

^a^Percentages may not add to exactly 100 due to rounding.

^b^Year 12 is the final year of high school in the Australian education system.

^c^Socio-Economic Indexes For Areas (SEIFA).

^d^Based on postal/zip code. Percentiles are the rankings within Victoria, with a percentile of ≤25 being the most disadvantaged quartile.

### Criterion Validity of the International Physical Activity Questionnaire and Modified Active Australia Survey

**Figure 2:**
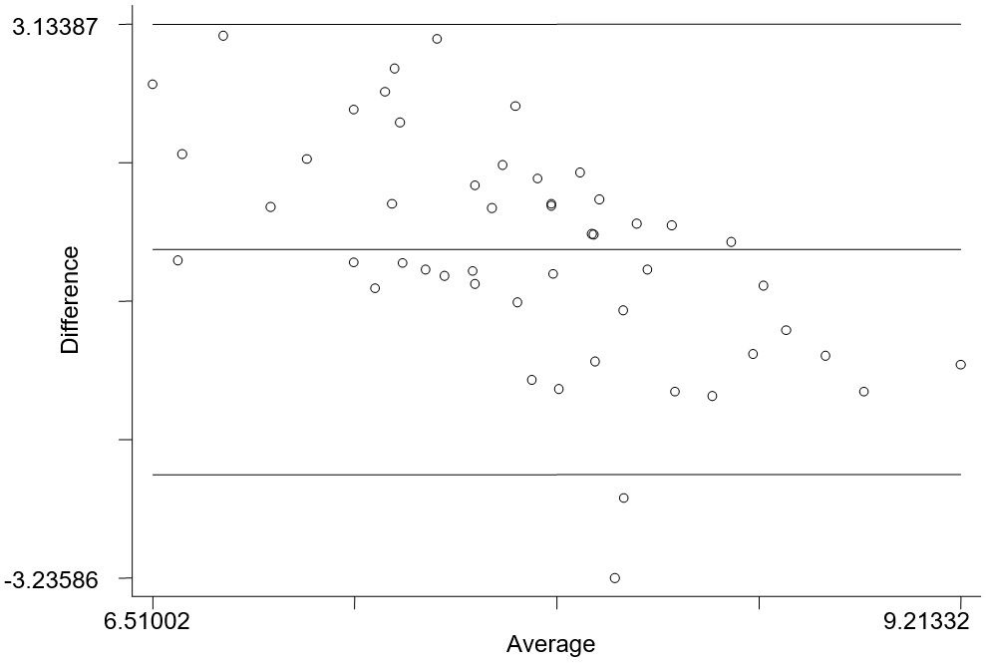
Bland-Altman plot of natural log transformed score showing the difference between IPAQ and SWA, plotted against the mean. Note: the lines represent the limits of agreement (95%) and average difference between the two variables.

There was no significant correlation observed between follow-up IPAQ scores and SWA minute-by-minute continuous data (*r*=.10, *P*=.48) and follow-up IPAQ scores and SWA blocked continuous data (*r*=.07, *P*=.63). Both distributions are scattered and no linearity is observed. The negative slope of the band in the Bland-Altman plot reveals a tendency for IPAQ to give higher scores than the SWA (see Figure 2).

**Figure 3:**
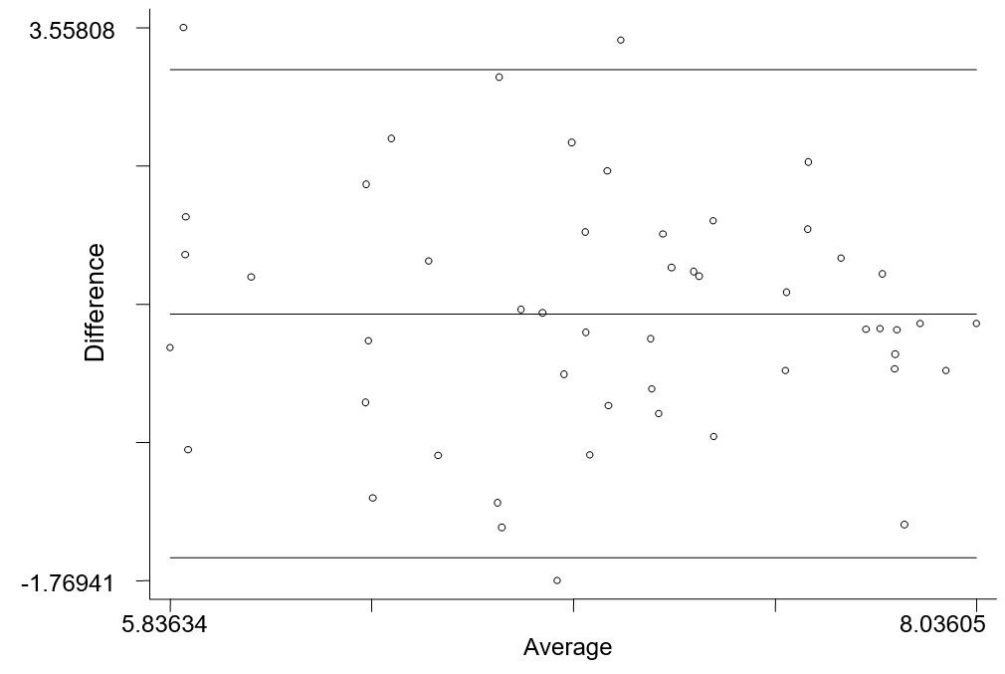
Bland-Altman plot of natural log transformed score showing the difference between MAAS and SWA, plotted against the mean. Note: the lines represent the limits of agreement (95%) and average difference between the two variables.

No significant correlations were observed between follow-up MAAS scores and SWA minute-by-minute continuous data and SWA blocked continuous data (*r*=.02, *P*=.88; *r*=.05, *P*=.72, respectively; Figure 3).

### Acceptability of the SenseWear Armband

Table 2 summarizes participants’ experiences using the SWA. The majority of participants reported a positive experience, with more than half (29/52, 56%) reporting the SWA as not painful to wear, and just under a third (15/52, 29%) reporting not feeling at all self-conscious wearing it. A large proportion of participants (>75%) did not stop wearing the SWA due to feeling self-conscious about wearing it, finding it uncomfortable or painful to wear, interfering with sleep, being prohibited from wearing it, or concerns of getting it wet. A total of 67% (35/52) of participants preferred using the SWA over completing a survey about their physical activity, and 29% (15/52) were interested in using the SWA daily on an ongoing basis. The majority of participants (34/52, 65%) reported no increase in physical activity because of wearing the SWA.

**Table 2:** Participants’ experiences of using the SenseWear Armband (n=52)^a^.

Statements from the questionnaire	Agreement scale (1=completely false to 5=completely true), n (%)					Median (IQR^b^)
	1	2	3	4	5	
I often felt self-conscious wearing the activity monitor.	15 (29)	11 (21)	13 (25)	11 (21)	2 (4)	2.5 (1.0-3.3)
I often found the activity monitor uncomfortable to wear.	10 (19)	17 (33)	14 (27)	7 (13)	4 (8)	2 (2-3)
I often found the activity monitor painful to wear.	29 (56)	13 (25)	7 (13)	2 (4)	1 (2)	1 (1-2)
I often felt proud to be seen wearing the activity monitor.	7 (13)	17 (33)	18 (35)	9 (17)	1 (2)	3 (2-3)
I often did not wear the activity monitor because I felt self-conscious about being seen wearing it.	43 (83)	7 (13)	1 (2)	1 (2)	0 (0)	1 (1-1)
I often did not wear the activity monitor because it was uncomfortable or painful.	42 (81)	7 (13)	3 (6)	0 (0)	0 (0)	1 (1-1)
I often did not wear the activity monitor overnight because it interfered with my sleep.	40 (77)	5 (10)	4 (8)	2 (4)	1 (2)	1 (1-1)
I often did not wear the activity monitor during exercise because it would have got wet (eg, while swimming, or because I was walking in the rain).	37 (71)	7 (13)	2 (4)	3 (6)	3 (6)	1 (1-2)
I often did not wear the activity monitor because it was prohibited (eg, not allowed to wear it during netball, or in my workplace).	46 (88)	3 (6)	0 (0)	2 (4)	1 (2)	1 (1-1)
I exercised more than I otherwise would have, because of wearing the activity monitor.	34 (65)	13 (25)	3 (6)	1 (2)	1 (2)	1 (1-2)
I would prefer to record my physical activity for the last 7 days by completing a survey rather than wearing the activity monitor for 7 days.	35 (67)	7 (13)	7 (13)	2 (4)	1 (2)	1 (1-2)
I would like to wear the activity monitor everyday if I could get real-time feedback of my physical activity and calories burned.	5 (10)	4 (8)	13 (25)	15 (29)	15 (29)	4 (3-5)

^a^There were 52 respondents due to missing data on these items for 2 participants.

^b^Interquartile range (IQR).

### Concurrent Validity of the Modified Active Australia Survey

Both categorical and continuous measures of the follow-up MAAS and IPAQ showed moderate agreement. Based on categorical data, 65% of cases (34/52) were in agreement, which corresponds to moderate agreement between 2 measures (kappa=.48, *P*<.001) (see Table 3).

**Table 3:** Categorical outcomes from MAAS^a^and IPAQ^b^follow-up questionnaires.

		IPAQ, n (%)			
		Low (n=17)	Moderate (n=16)	High (n=19)	Total (n=52)
**MAAS, n (%)**					
	Low	13 (76)	3 (19)	1 (5)	17 (33)
	Moderate	4 (24)	8 (50)	5 (26)	17 (33)
	High	0 (0)	5 (31)	13 (68)	18 (35)
	Total	17 (100)	16 (100)	19 (100)	52 (100)

^a^Modified Active Australia Survey (MAAS).

^b^International Physical Activity Questionnaire (IPAQ).

**Figure 4:**
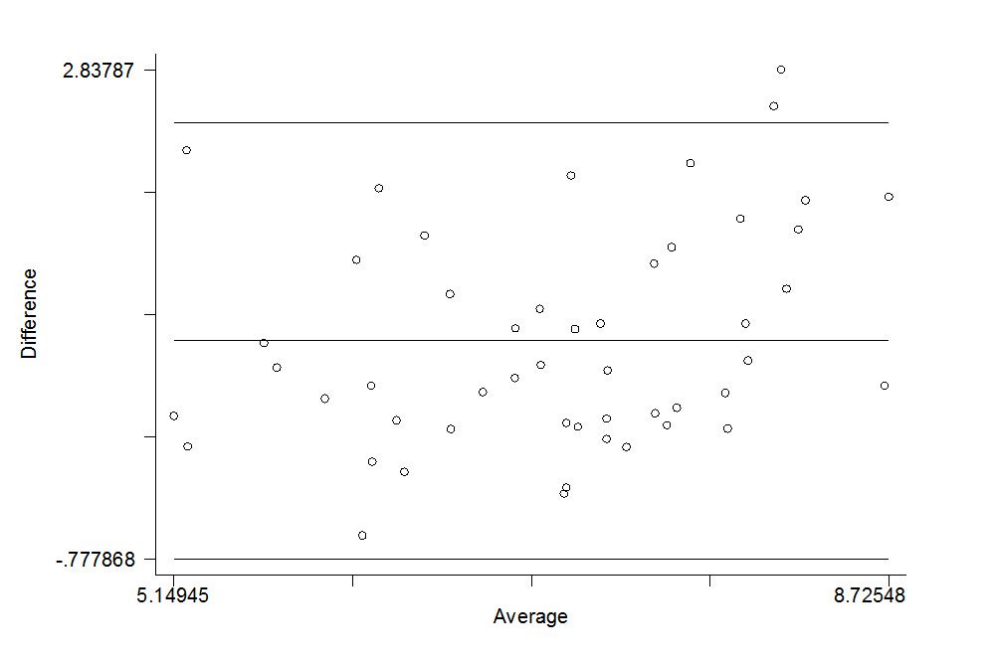
Bland-Altman plot of natural log transformed score showing the difference between follow-up IPAQ and MAAS, plotted against the mean. Note: the lines represent the limits of agreement (95%) and average difference between the two variables.

The results are similar when analyzed using continuous data with a canonical correlation of .69 (*P*<.001) and are in agreement with no visible consistent bias between 2 scores (limits of agreement are between -0.78 and 2.45; Figure 4).

### Test-Retest Analysis of the Modified Active Australia Survey

The median interval between completion of baseline and follow-up questionnaires was 11.5 days (IQR 9.0-14.5). In analysis of test-retest reliability of the MAAS, 2 participants were excluded as they reported increasing their activity levels as a result of using the SWA. There was poor agreement between baseline and follow-up MAAS categorical scores (kappa=.193, *P*=.03; Table 4).

**Table 4:** Categorical outcomes from baseline and follow-up MAAS^a^.

		Follow-up MAAS category, n (%)			
		Low (n=17)	Moderate (n=17)	High (n=18)	Total (n=52)
**Baseline MAAS category, n (%)**					
	Low	11 (65)	5 (29)	2 (11)	18 (35)
	Moderate	4 (24)	5 (29)	8 (44)	17 (33)
	High	2 (12)	7 (41)	8 (44)	17 (33)
	Total	17 (100)	17 (100)	18 (100)	52 (100)

^a^Modified Active Australia Survey (MAAS).

**Figure 5:**
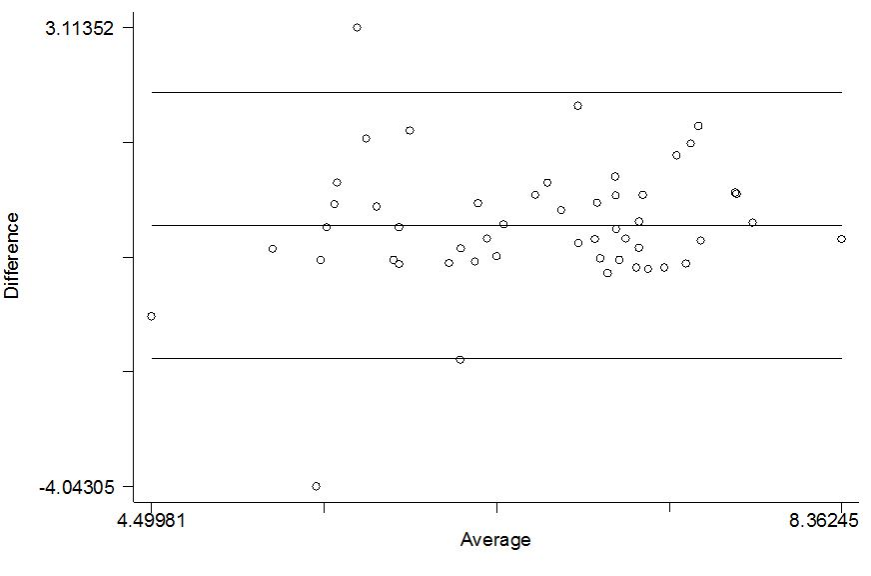
Bland-Altman plot of natural log transformed score showing the difference between baseline and follow-up MAAS, plotted against the mean. Note: the lines represent the limits of agreement (95%) and average difference between the two variables.

Canonical correlation yielded a correlation coefficient of .44 (*P*=.001). To show the differences in score as a function of the mean score, a Bland-Altman plot was constructed of the naturally logged baseline and follow-up MAAS scores (Figure 5). There was a mean difference of 0.03 (95% limits of agreement -2.04 to 2.10).

## Discussion

### Principal Findings

This study examined clinimetric properties of 3 physical activity assessment tools in young Australian women. The results showed that there was no significant correlation between SWA and IPAQ continuous data, either minute-by-minute or blocked SWA data. Comparison of the SWA and MAAS showed the same finding. The SWA tended to record lower scores than IPAQ and MAAS, suggesting participants tended to overreport their amount of physical activity. This effect is likely larger than shown in the results, as the SWA also captures incidental exercise, whereas IPAQ and MAAS do not. The amount of overreport is therefore likely larger and more substantial than observed. This finding of participant overreporting when compared to an objective monitoring device is consistent with findings from the much larger National Health And Nutrition Examination Survey (NHANES), which collected data from males and females aged 20 to 80+ years [21]. Physical activity monitors also have limitations in capturing all activity, and so may give an underreport of total activity which contributes to the differences between the SWA and questionnaire data.

Overall, the acceptability of the SWA was high. Notably, the majority of participants preferred to use the SWA rather than self-report their physical activity. A small number of participants (3/52, 6%) experienced localized skin reactions to the monitor, a known rare complication due to metal allergy. Despite this, adherence to SWA use was high and participant responses to the feedback questions indicated that the SWA is both acceptable and favored by young Australian women. While adherence to the SWA was high, it is not known whether repeated use would give similar adherence levels. It may be that the young women found the SWA to be a novelty initially and so were pleased to wear it for 7 days, but that enthusiasm may wane with repeated use. Given that studies often require participants to collect physical activity data more than once over an extended period, it would be important to ascertain young women’s preference for the SWA or questionnaires with repeated use.

According to Scheers et al [11], participants ideally should have at least 5 monitoring days, including 2 weekend days. However, for our analyses participants who had at least 4 monitoring days, including 2 weekend days, were included. This decision was made due to participant numbers. A larger cohort size would have allowed the conditions of Scheers et al to be applied, without losing too many participants from analysis. This is a limitation of this study which needs to be considered in interpreting the findings. It is possible that the days the participants wore the SWA were their most or least active days of the week, which would give an overestimation or underestimation of their total weekly activity, respectively. However, the agreement of our findings with the NHANES study [21], as discussed above, gives us greater confidence in them, despite this limitation.

Since this study was conducted, there has been a large increase in the availability and range of devices available for objectively monitoring physical activity and other body parameters, such as sleep and calorie intake. Many of these have the advantage of being considerably less costly and more lightweight than the SWA, and future studies would need to consider the advantages of using the SWA compared with these newer devices. However, our finding that a monitoring device tends to record lower levels of physical activity than self-reported questionnaires is likely applicable to other devices. We would expect that these devices would also share the acceptability to young women found for the SWA in this study, and that they may indeed be more acceptable due to their smaller and more lightweight design.

The test-retest reliability of the MAAS was moderate when using continuous outcomes. This is consistent with data from a study using MAAS in 159 middle-aged Australian women [13]. However, test-retest reliability was poor when using categorical outcomes. A possible explanation for lack of strong agreement is that participants were not excluded from this analysis if they indicated their activity levels had changed due to factors other than wearing the SWA. In order to be excluded from this analysis, participants had to indicate that their level of activity had increased due to wearing the SWA. Participants were not asked about increased or decreased physical activity due to factors other than the SWA—such as illness, holidays, or work commitments—so participants who experienced this were not excluded from this analysis.

Comparisons between the MAAS and IPAQ questionnaires showed moderate agreement for both categorical and continuous data. There was an overall tendency for the IPAQ to give a higher score, which may be due to the more detailed questions on different domains of activity contained in the IPAQ. Given the moderate level of agreement between MAAS and IPAQ and the moderate test-retest reliability when using continuous outcomes, MAAS may be a suitable alternative to IPAQ to assess total physical activity score, due to its shorter length and consequent reduction in participant burden. The analyses performed in this study were only for the total physical activity scores obtained using each instrument. Further analyses of the correlation between domains of physical activity would be useful in assessing comparison of the instruments.

A limitation of this study is the relatively small sample size and this needs to be considered in interpreting the results. A larger study would allow our findings to be further tested. The recruitment method used in this study has been shown to recruit a representative sample of young women, therefore we are confident our sample was not overly selective [16].

### Conclusions

Physical activity is an important contributor to health and the prevention of disease, and its study requires reliable and valid measures which can be administered to individuals. This study showed that the MAAS has moderate agreement with the IPAQ, and as such may be a suitable alternative in some situations. Test-retest analysis of the MAAS did not give strong results; however, this is potentially explained by a limitation of the study in the omission of a question on changes in activity levels. This study showed that young women tended to overreport their physical activity when compared to the SWA monitor, suggesting the SWA may be a more accurate tool which overcomes some of the limitations of self-report instruments. This finding, combined with the high acceptability of the device to young women, suggests the SWA, and perhaps other such monitors, may be a better option for measuring physical activity for both researchers and research participants.

## Abbreviations

GSK: GlaxoSmithKline
HPV: human papillomavirus
IPAQ: International Physical Activity Questionnaire
IQR: interquartile range
MAAS: Modified Active Australia Survey
MET: metabolic equivalent of task
MSD: Merck Sharp & Dohme
NHANES: National Health And Nutrition Examination Survey
SEIFA: Socio-Economic Indexes For Areas
SWA: SenseWear Armband
VACCINE: Vaccine Against Cervical Cancer Impact aNd Effectiveness
VIVIANE: Human PapillomaVIrus Vaccine Immunogenicity ANd Efficacy
YFHI: Young Female Health Initiative
